# Conditioned Medium of Human Menstrual Blood-Derived Endometrial Stem Cells Protects Against MPP^+^-Induced Cytotoxicity *in vitro*

**DOI:** 10.3389/fnmol.2019.00080

**Published:** 2019-04-05

**Authors:** Han Li, Badrul Hisham Yahaya, Wai Hoe Ng, Narazah Mohd Yusoff, Juntang Lin

**Affiliations:** ^1^Advanced Medical and Dental Institute, Universiti Sains Malaysia, Penang, Malaysia; ^2^Stem Cell and Biotherapy Engineering Research Center of Henan, College of Life, Science and Technology, Xinxiang Medical University, Xinxiang, China; ^3^Henan Joint International Laboratory of Stem Cell Medicine, College of Biomedical Engineering, Xinxiang Medical University, Xinxiang, China

**Keywords:** conditioned medium, inflammation, MPP^+^, MSCs, Parkinson’s disease, ROS, SH-SY5Y

## Abstract

Mesenchymal stem cells (MSCs) showed the potential to treat Parkinson’s disease (PD). However, it is unknown whether the conditioned medium of human menstrual blood-derived endometrial stem cells (MenSCs-CM) has the function to alleviate syndromes of PD. In this study, human neuroblastoma SH-SY5Y cells were exposed to neurotoxicant 1-methyl-4-phenylpyridinium (MPP^+^) for inducing a range of response characteristics of PD. After culturing this cell model with 24 h/48 h collected MenSCs-CM for different days, cell viability, pro-inflammation cytokines, mitochondrial membrane potential (ΔΨm), oxidative stress, and cell apoptosis were detected. Finally, protein assay was performed to detect 12 kinds of neurotrophic factors inside MenSCs-CM. Our results showed that MPP^+^ caused SH-SY5Y cell viability reduction as an increasing dose and time dependent manner. MPP^+^ treatment resulted in inflammation, mitochondrial dysfunction, reactive oxygen species (ROS) production accumulation, and apoptosis of SH-SY5Y at its IC50 concentration. Forty-eight hours-collected MenSCs-CM and culturing with the MPP^+^-treated SH-SY5Y for 2 days are the optimized condition to increase cell viability. Besides, MenSCs-CM was efficacious against MPP^+^ induced inflammation, ΔΨm loss, ROS generation, and it could significantly decrease cells numbers in late apoptosis stage. What’s more, protein assay showed that MenSCs-CM contained various neuroprotective factors. Our study provided the first evidence that MenSCs-CM has a protective effect on MPP^+^-induced cytotoxicity in various aspects, and firstly showed that MenSCs can release at least 12 kinds of neurotrophic factors to medium, which may contribute to the protective function of MenSCs-CM to treat PD. This research enlightening that MenSCs-CM is beneficial in the therapy for PD and probably also for other neurodegenerative diseases.

## Introduction

Parkinson’s disease (PD) is the second most common age-related neurodegenerative disease, which is characterized by dopamine neurons loss in substantial nigra, Lewy body formation, and dopamine depletion in striatum (Kim et al., 2017; Yasuhara et al., 2017). Although the etiopathogenesis of PD has not been fully unraveled, accumulating evidence suggest that neuroinflammation, mitochondrial dysfunction, oxidative stress, and dopamine neurons apoptosis are closely associated with the progression of PD (Dauer and Przedborski, 2003; Canet-Aviles et al., 2014; Kim et al., 2017).

Over the last decade, mesenchymal stem cells (MSCs), derived from bone marrow, adipose, umbilical cord and endometrium, were shown to improve animal PD model after brain stereotaxic transplantation (McCoy et al., 2008; Wolff et al., 2011, 2015; Campeau et al., 2014; Kumar et al., 2016). These studies showed that transplanted MSCs could survive in PD mice striatum, differentiate into dopaminergic neuron-like cells, increase dopamine/dopamine metabolite concentrations and promote neurological functional recovery (McCoy et al., 2008; Wolff et al., 2011, 2015; Campeau et al., 2014; Kumar et al., 2016). Nonetheless, the differentiation efficiency of MSCs to dopaminergic neurons *in vivo* was low, with only 0.01% dopaminergic neurons originated from MSCs (Wolff et al., 2015). Besides, the differentiation ability of MSCs was challenged in other studies, for example, bone marrow and umbilical cord matrix derived MSCs did not change their initial phenotype after engraftment and failed to differentiate into dopaminergic neurons in mice brain following transplantation (Kang et al., 2013; Neirinckx et al., 2013). Therefore, we hypothesize that MSCs can improve PD through paracrine secreting some trophic factors to provide local neuroprotective and neurotrophic, for example, reducing cell apoptosis, exerting anti-oxidative effects and secreting cytokines that can mediate immune response such as anti-inflammatory. Thus, conditioned medium collected from MSC culture *in vitro* is suggested to have therapeutic potential in improving PD symptom through the release of various neurotrophins and cytokines.

In comparison with MSCs as mentioned above, human menstrual blood-derived endometrium stem cells (MenSCs) can easily be obtained noninvasively and collected periodically, which makes it a valuable resource for cell-based therapies (Liu et al., 2018). Furthermore, there is no preclinical or clinical research on the application of MenSCs for treating PD. By constructing an *in vitro* SH-SY5Y PD cell model induced by neurotoxin 1-methyl-4-phenylpyridinium (MPP^+^), we sought to investigate if MenSCs could improve MPP^+^-induced cytotoxicity by paracrine secretion. We collected conditioned medium from MenSCs at different days (MenSCs-CM) in this study. MPP^+^-treated SH-SY5Y cells were cultured in MenSCs-CM for different days. The effect of MenSCs-CM was assessed based on cell viability, inflammatory response, mitochondrial membrane potential, oxidative stress, and apoptosis. Finally, protein assay was performed to analyze the neurotrophic factors secreted by MenSCs.

## Materials and Methods

### Ethics and Reagents

The procedure of collecting human samples was carried out in accordance with the recommendations from the human research ethics committee of Universiti Sains Malaysia (Code: USM/JEPeM/16070230). All subjects were given written informed consent in accordance with the Declaration of Helsinki.

Chemicals were of analytical grade and purchased from Sigma-Aldrich Corp (Saint Louis, MO, USA) and reagents for cell culture were bought from Gibco (Grand Island, NY, USA), unless otherwise specified.

### MenSCs Isolation and Culture

The MenSCs were isolated and cultured as described previously with minor modifications (Liu et al., 2018). Briefly, approximately 5 mL menstrual blood was collected from healthy women donors using menstrual cups (Diva Cup, USA) during the first few days of the menstrual period cycle. An equal volume of blood sample was added to Ficoll-Paque media (GE Healthcare, Sweden) carefully and centrifuged at 400 *g* for 30 min at room temperature. Following density gradient centrifugation, plasma and platelets in the upper layer were removed using a pipette and mononuclear cell layer remained undisturbed at the interface. The mononuclear cell layer was transferred to a sterile centrifuge tube and washed twice with PBS. Cell pellets were grown in Dulbecco’s modified Eagle’s high glucose (DMEM-HG) medium supplemented with 100 μ/mL penicillin, 100 mg/mL streptomycin, and 10% FBS (Gibco, South America). Cells were kept at 37°C in a humidified atmosphere with 5% CO_2_. Media was changed after 24 h to remove all floating cells, followed by regular media changes every 3 days.

### Immunophenotyping Analysis

Cell surface markers of passage 3 MenSCs were determined by direct immunofluorescence staining and analyzed by flow cytometer. All antibodies and blocking buffer were purchased from Becton, Dickinson and Company (NJ, USA) unless otherwise specified. Cells were harvested by using TrypLE Express (Gibco, Denmark) and the cell pellet was achieved by centrifuge at 300 *g* for 5 min (Sigma, Saint Louis, MO, USA). Pellet was resuspended in blocking buffer and cell concentration was adjusted to be 10^7^/mL. Cells were aliquoted to different tubes, 100 μL/tube. Next, respective antibodies for identifying MSC surface markers such as CD19-APC, CD34-PE, CD45-PerCP, CD73-APC, CD90-APC, CD105-APC, and relative isotype control antibodies were added to relative tubes. In all experiments, the corresponding isotype-matched, non-reactive fluorochrome-conjugated antibodies were used as negative controls. Tubes were kept on ice for 30 min in dark. Cells were washed once with blocking buffer to remove uncombined antibody. Finally, cells were resuspended in 500 μL blocking buffer. Data (10,000 events) were collected using a FACS Calibur (BD Biosciences, CA, USA) and analyzed using FlowJo version 10 software.

### Multilineage Differentiation Assays

Adipogenesis, osteogenesis, and chondrogenesis differentiations were performed using respective differentiation kits (StemPro, Thermo Fisher Scientific, CA, USA).

To test if MenSCs were able to differentiate into adipocytes and osteoblasts, sub-confluent MenSCs at P3 were treated with adipogenesis/osteogenesis differentiation medium, respectively. The medium was changed every other day and undifferentiated controls were cultured concurrently in the growth medium. On day 14, the lipid droplets of differentiated cells in adipogenesis differentiation medium was stained with Oil Red O, with nuclei counterstained by hematoxylin. For osteogenesis assay, after 21 days of induction, Alizarin Red S solution (PH4.2) was used to stain mineralization nodes. The staining was observed and captured with an inverted microscope (Olympus).

For chondrogenesis assay, the cell concentration of passage 3 MenSCs was adjusted to be 1.6 × 10^7^ cells/mL. From there, 5 μL of cell suspension was dropped in the center of 24-well plate wells to generate micromass cultures. After cultivation for 2 h under high humidity conditions, chondrogenesis media/growth media (for control) was added carefully in wells without disturbing the micromass, and the medium was changed every 2–3 days. After 14 days, chondrogenesis differentiation was confirmed by Alcian blue staining. Images were captured with an inverted microscope (Olympus).

### Preparation of MenSC Conditioned Medium

When MenSCs were 70%–80% sub-confluent in a T75 flask, the growth medium was replaced with 20 mL serum-free DMEM-HG medium (containing 100 μ/mL penicillin, 100 mg/mL streptomycin). Culture medium was collected after 24 h and 48 h incubation, centrifuged at 600 *g* for 5 min and passed through a 0.22 μm filter, which was termed as a conditioned medium of MenSCs (MenSCs-CM). The aliquoted MenSCs-CM was stored at −80°C till use. All *in vitro* experiments were performed using passage 3–5 MenSCs-CM.

### Preparation of Exosomes and Exosomes-Depleted MenSCs-CM

MenSCs-derived exosomes (MenSCs-Exo) were isolated freshly from the 48 h collected MenSCs-CM. The medium was centrifuged at 10,000 *g* for 30 min at 4°C (Hettich Universal 320R, Germany). The supernatant was transferred to an ultracentrifuge tube (Beckman Coulter, USA) and centrifuged at 100,000 *g* for 130 min at 4°C (Beckman Coulter 70 Ti, USA). The supernatant was carefully transferred to new tubes and kept at 4°C for later use, and this was termed exosomes-depleted MenSCs-CM (EDM). The sediment was washed twice with cold PBS by centrifugation at 100,000 *g* for 130 min at 4°C. PBS was removed carefully using Pasteur suction. Then 200 μL cold PBS was added to dissolve the attached on the wall of the ultracentrifuge tube. The exosomes were aliquoted and kept in −80°C for use within 1 week.

### Neuroblastoma Cell Culture and Drug Treatment

Neuroblastoma cell line SH-SY5Y was obtained from the American Type Culture Collection (ATCC, Manassas, USA). Cells were maintained in DMEM-HG medium supplemented with 10% heat-inactivated fetal bovine serum, 100 μ/mL penicillin, and 100 μg/mL streptomycin under 95% humidified air containing 5% CO_2_ at 37°C.

1-Methyl-4-phenylpyridinium iodide (MPP^+^) was prepared freshly every time before use by dissolving in serum free DMEM-HG medium. SH-SY5Y was seeded as 15,000 cells/well in 96-well plate. After 80% confluent, cells were treated with different concentrations of MPP^+^ (0.5 mM, 1 mM, 2 mM, 3 mM, 4 mM, 5 mM, and 6 mM) at different time points (6 h, 12 h, 24 h, 36 h, 48 h, and 72 h).

### Treatment With MenSCs-CM/MenSCs-Exo/EDM/MenSCs

After treatment with MPP^+^ drug for desired days, the medium was removed and washed twice with serum-free DMEM-HG medium. For control group and MPP^+^+DMEM group, serum-free DMEM-HG medium was added; for MPP^+^+MenSCs-CM group, 24 h/48 h-collected MenSCs-CM was added, respectively; for MPP^+^+MenSCs-Exo group, 5 μg/mL, 50 μg/mL, 100 μg/mL, and 200 μg/mL MenSCs-Exo was added, respectively; for MPP^+^+EDM group, EDM was added. After another 1–3 days cultivation, cell viability was detected using the protocol described below.

Indirect co-culture system was developed by culturing Passage 3 MenSCs on 24-well transwell insert. Growth medium of sub-confluent cells in the insert were then replaced by 500 μL serum free medium and indirectly co-culture with SH-SY5Y for 1–3 days. Cell viability was detected at indicated time points using the protocol described below.

### Cell Viability Assay

After treated with MPP^+^ or cultured with MenSCs-CM//MenSCs-Exo/EDM/MenSCs, cell viability of SH-SY5Y cells was assessed *via* PrestoBlue assay (Invitrogen, USA) according to the manufacturer’s protocol with some modifications. Briefly, at the indicated time point, 10 μL PrestoBlue was added directly to cells in a 96-well plate. Background fluorescence was corrected to blank wells containing only medium without cells. Cells were incubated for 30 min at 37°C in dark and then fluorescence was read at Ex544 nm/Em590 nm using a FLUOstar Omega microplate reader (BMG Labtech, Offenburg, Germany). The cell viability was calculated according to the following formula: Cell viability (%) = [(fluorescence of treatment group − blank/fluorescence of control group − blank)] × 100%.

### RNA Isolation and Real-Time RT-PCR

Total RNA was isolated using an RNA isolation kit (Qiagen, Hilden, Germany) according to the manufacturer’s instructions. cDNA was synthesized using cDNA synthesis kit (Bioline, London, UK) according to manufacturer’s protocols. Genes detected here included cyclooxygenase (*COX-2*), interleukin-1β (*IL-1β*), interleukin-6 (*IL-6*), inducible nitric oxide synthase (*iNOS*), tumor necrosis factor-α (*TNF-α*), peroxiredoxin-1 (*PRDX-1)*, thioredoxin (*TXN)*, heme oxygenase-1 *(HMOX-1)*, B-cell lymphoma/leukemia-2 associated agonist of cell death (*Bad*), B-cell lymphoma/leukemia-2 associated X protein (*Bax*), and B-cell lymphoma-extra large (*Bcl-xl*). Real-time PCR was performed using QuantiNova^TM^SYBR^®^ GreenPCR kit (Qiagen, Hilden, Germany) and glyceraldehyde-3-phosphate dehydrogenase (*GAPDH*) was used as an internal standard. Briefly, real-time PCR amplification was performed for all genes under the following conditions on a Step One Plus Real-Time PCR System (Applied Biosystems, CA, USA): 2 min at 95°C for initial denaturation; followed by 40 cycles of 5 s at 95°C, and 10 s at 60°C; melt curve stage 15 s at 95°C, and 1 min at 70°C; denaturation step, 15 s at increment of +0.3°C until 95°C. Twenty microliter reaction mixtures contained 10 μL 2× SYBR Master Mix, 0.7 μM primers pair (Supplementary Table S1; Bioneer, Daejeon, South Korea), 2 μL ROX reference dye, 50 ng cDNA, and RNase-free water. All samples were run in parallel triplicates and at least three independent experiments were performed. Expression of the gene of interest was normalized to housekeeping gene GAPDH. The relative expression of each gene was calculated by formula 2^–ΔΔCt^.

### Measurement of Mitochondrial Membrane Potential (ΔΨm)

Rhodamine123 (sc-208306, Santa Cruz, Dallas, TX, USA) was used to monitor mitochondrial membrane potential (ΔΨm) by flow cytometry. Briefly, cells were harvested at the indicated time points and washed once with supplement buffer (PBS+10% FBS). Ten microgram per milliliter Rhodamine123 was added in cell suspension and incubated at 37°C in dark for 30 min. After washed once with PBS, cells were resuspended in 500 μL supplement buffer and transferred to flow cytometry tubes. Fluorescence intensity was measured by flow cytometry (Calibur, BD, CA, USA) using channel FL1. At least 10,000 events were analyzed per sample and three independent experiments were done for replication. Data were analyzed using FlowJo version10 software.

### Detection of Intracellular ROS Levels

Intracellular superoxide anion production was measured using Dihydroethidium (DHE, sc-204724A, Dallas, TX, USA) by flow cytometry or observed under fluorescence microscope.

For flow cytometry, the cell concentration was adjusted to be 1 × 10^6^/mL. Ten nanogram per microliter DHE was added into cells and was incubated at 37°C in dark for 30 min. Fluorescence intensity was measured by flow cytometry (Calibur, BD, NJ, USA) using channel FL3. At least 10,000 events were analyzed per sample and three independent experiments were performed for replication. Data were analyzed using FlowJo version 10 software.

For fluorescence microscope observation, SH-SY5Y cells were seeded in a 24-well plate. After treated with MPP^+^ for the indicated time, the medium was removed and washed twice with PBS. Supplement buffer (PBS+10% FBS) containing 10 ng/μL DHE was added in wells and incubated at 37°C in dark for 30 min. Then cells were observed directly under a fluorescence microscope (Olympus TH4-200, Leica camera). Ten images were captured randomly under 40× magnification from each well, and triplicate wells each group. Fluorescence intensity was quantified using Image J software. The value of % fluorescence intensity was normalized to the control group.

### Apoptosis Assay

Apoptosis assay was performed using the Annexin V-FITC Apoptosis Detection Kit II (51-6710AK, BD Pharmingen, NJ, USA) according to manufacturer’s protocol. Briefly, 1 × 10^6^ SH-SY5Y cells were harvested and washed once with cold PBS. Cells were re-suspended in 100 μL 1× binding buffer, followed by adding 5 μL Annexin V and 5 μL propidium iodide (PI). Then cells were kept on ice for 15 min in dark. Apoptosis was analyzed by flow cytometry (Calibur, BD Biosciences, NJ, USA). A minimum of 10,000 events was analyzed per sample. Three independent experiments were done for replications.

### Protein Assay

A protein array kit (Ray Biotech Inc., Norcross, GA, USA) was used to detect 12 different components of neurotrophic factors in MenSCs-CM according to the manufacturer’s instructions. The neurotrophic factors included artemin (ARTN), brain-derived neurotrophic factor (BDNF), conserved dopamine neurotrophic factor (CDNF), glial cell-derived neurotrophic factor (GDNF), hepatocyte growth factor (HGF), insulin-like growth factor-1 (IGF-1), mesencephalic astrocyte-derived neurotrophic factor (MANF), nerve growth factor (NGF), neurotrophin-3 (NT-3), neurotrophin-4/5 (NT-4/5), neurturin (NTN), and persephin (PSPN). Signals were scanned by InnoScan 300 Microarray Scanner (Innopsys, Carbonne, France). Data were presented as mean ± standard deviation (SD; *N* = 3).

### Statistical Analysis

All data were in mean ± SD from at least three independent experiments. Fluorescence intensity was measured by Image Pro-Plus 6.0 version software (Media Cybernetics, USA). Statistical analyses included Student’s *t*-tests for two groups, and comparisons among three groups were analyzed by one-way ANOVA, followed by Bonferroni’s *post hoc* test. By convention, a *p*-value < 0.05 was considered statistically significant.

## Results

### MenSCs Characterization

**Figure 1:**
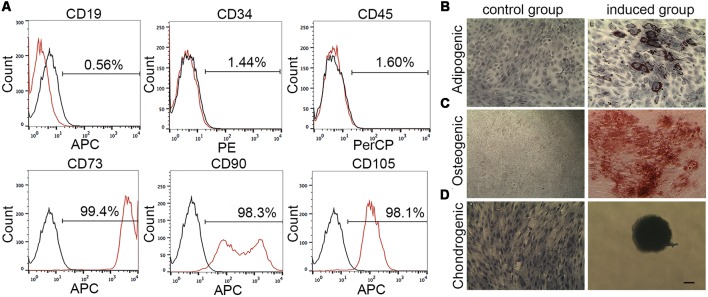
Menstrual blood-derived endometrium stem cells (MenSCs) characterization by flow cytometry and mesodermal differentiation assay. **(A)** The expression of MenSCs surface markers was detected by flow cytometry. Black lines stand for isotype controls and red lines represent for CD markers expression. **(B)** MenSCs underwent adipogenesis. Cell nucleus was stained by hematoxylin. **(C)** MenSCs underwent osteogenesis. **(D)** MenSCs underwent chondrogenesis. Cell nucleus was stained by hematoxylin. Scale bar in **(D)** is also suitable for **(B,C)**.

The immunophenotype of passage 3 MenSCs was characterized by flow cytometry, according to the guideline from International Society for Cellular Therapy. MenSCs expressed typical MSCs surface markers (*n* = 4), including abundant positive for CD73 (99.04%–99.53%), CD90 (94.05%–98.61%), and CD105 (95.04%–98.16%) and negative for CD19 (0.12%–0.57%), CD34 (1.24%–1.98%), and CD45 (1.2%–1.8%). Figure 1A is one representative result of four biological replications.

Figures 1B–D showed that MenSCs had capacities to differentiate into adipocytes, osteoclasts and chondrocyte, respectively. In Figure 1B; in the induced group, the differentiation into adipocytes was revealed by the formation of lipid droplets stained by Oil Red O after 14 days of induction. The lipid droplets appeared red color grape-like clusters of small perinuclear vesicles. In Figure 1C, the differentiation into osteoblasts was demonstrated by Alizarin Red S staining of the red color mineralization nodes after 21 days of induction. Control cells without being treated with adipogenesis/osteogenesis medium failed to demonstrate any of these indicators. In Figure 1D, cell micromass without being treated with differentiation medium could migrate, proliferate and become confluent in wells after 14 days of culture. While, in the induced group, micromass was developed to cartilage spheroid, which exhibited an intensely blue color indicative of cartilage extracellular matrix monitored by Alcian blue staining.

### Effect of MPP^+^ on SH-SY5Y Cell Viability

**Figure 2:**
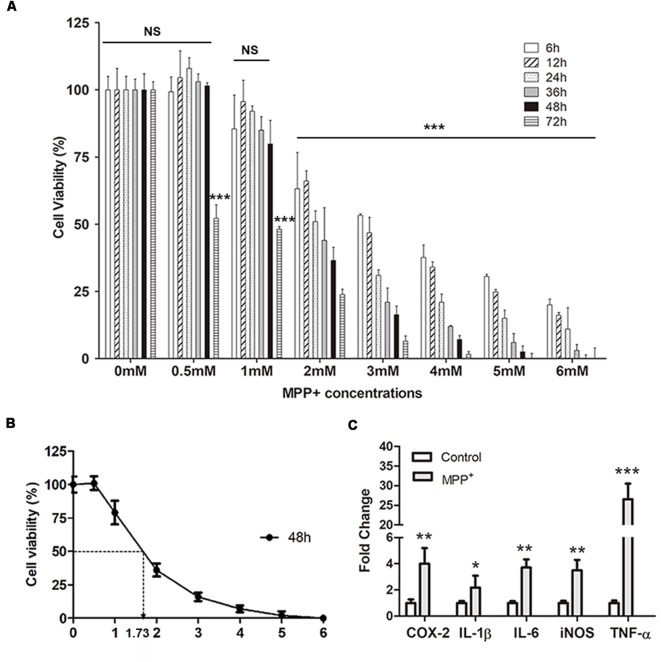
SH-SY5Y cell viability and inflammation assay. **(A)** Cell viability was analyzed after treated with different concentrations of MPP^+^ at different time points. **(B)** Polynomial regression analysis was performed to calculate IC50 of MPP^+^ at 48 h. **(C)** qRT-PCR was performed to detect mRNA expression of pro-inflammation factors after treated without/with 1.73 mM MPP^+^ for 2 days. Values were normalized to the values of control. Histograms are mean expression from three to five biological replicates. Error bars represent standard deviation (SD). Student *t*-test was applied to analyze the data. *p < 0.05, **p < 0.01, and ***p < 0.001 compared with control, NS, no significant difference.

Generally, after treated with different concentrations of MPP^+^ for different days, the cell viability of SH-SY5Y decreased as an increasing dose and time-dependent manner, except for treatment with the 0.5 mM MPP^+^ for 6 h, 12 h, 24 h, 36 h, and 48 h (Figure 2A). But there is no significant difference compared with control group (*p* > 0.05). After culturing 2–6 mM MPP^+^ with SH-SY5Y for 2–72 h, the cell viability significantly decreased compared with 0 mM group (***p < 0.001). Furthermore, there was a huge cell viability reduction during 1–2 mM at each time point (Figure 2A). IC50 (50% inhibitory concentration) of MPP^+^ was evaluated at 48 h by polynomial regression analysis (Figure 2B). The regression equation was *y* = 3.9041*X*2 − 41.683*X* + 110.47 and the correlation coefficient *R*^2^ was 0.97459. According to the equation, the IC50 of MPP^+^ at 48 h was 1.73 mM (Figure 2B). 1.73 mM MPP^+^ and exposure to SH-SY5Y cells for 48 h were used for the following studies.

### MPP^+^ Increases Inflammation Genes Expression

After treated with 1.73 mM MPP^+^ for 48 h, the mRNA expression of pro-inflammation genes *COX-2*, *IL-1β*, *IL-6*, *iNOS*, and *TNF-α* was quantified by qRT-PCR. The results showed that the expression of these genes was up-regulated after MPP^+^ treatment. After normalized to the value of control, the mRNA expression of *COX-2*, *IL-1β*, *IL-6*, *iNOS*, and *TNF-α* was 4.01 ± 1.2 (**p < 0.01), 2.17 ± 0.91 (*p < 0.05), 3.72 ± 0.61 (**p < 0.01), 3.5 ± 0.8 (**p < 0.01), and 26.55 ± 3.95 (***p < 0.001), respectively.

### MPP^+^ Elevates ROS Generation and Decreases ΔΨm in SH-SY5Y Cells

**Figure 3:**
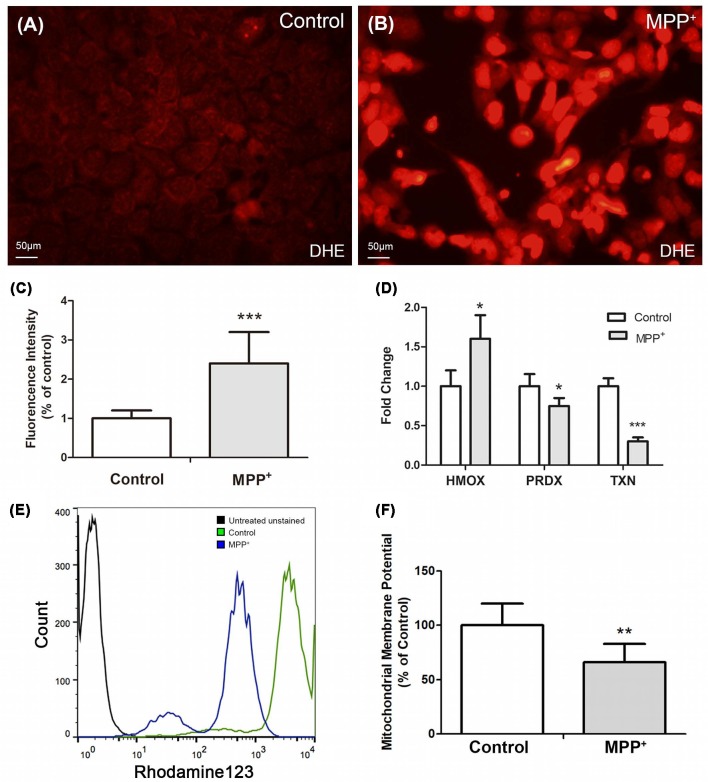
MPP^+^ induced reactive oxygen species (ROS) and decreased ΔΨm in SH-SY5Y cells. **(A)** Cells without were stained with DHE without exposure to MPP^+^. **(B)** Cells were stained with DHE after treated with 1.73 mM MPP^+^ for 48 h, and then imaged under a fluorescence microscope. **(C)** Fluorescence intensity of DHE was analyzed by image J software. **(D)** The mRNA expression of *HMOX-1*, *PRDX-1*, and *TXN* was analyzed by qRT-PCR. **(E)** Fluorescence intensity of Rhodamine123 was analyzed by flow cytometry. **(F)** Student *t*-test was performed to analyze the fluorescence intensity between control and MPP^+^ group. All Histograms are mean expression from three to five biological replicates. Values were normalized to the values of control. Error bars represent SD. *p < 0.05, **p < 0.01, and ***p < 0.001 compared with control.

DHE was used to stain reactive oxygen species (ROS), especially superoxide in SH-SY5Y cells. There was low level superoxide in control group cells (Figure 3A), while it increased profoundly after treated with MPP^+^ (Figure 3B), and there existed a significant difference of fluorescence intensity between two groups (Figure 3C). Furthermore, the expression of anti-oxidant genes *PRDX-1* and *TXN* was reduced, which suggested they played an important role in regulating MPP^+^-induced ROS generation (Figure 3D). Besides, the expression of anti-oxidant gene *HMOX-1* increased, although its increasing failed to inhibit ROS generation (Figure 3D).

Mitochondrial membrane potential (ΔΨm) is one of the vital parameters of mitochondrial function and an indicator of cell health. Depletion of ΔΨm suggests the loss of mitochondrial membrane integrity reflecting the initiation of the pro-apoptotic signal. After treated with 1.73 mM MPP^+^ for 48 h, the ΔΨm of SH-SY5Y cells significantly decreased compared with control group (Figures 3E,F).

### MPP^+^ Induces SH-SY5Y Cell Apoptosis Through Dysregulation and Regulation of Pro- and Anti-apoptosis Genes

**Figure 4:**
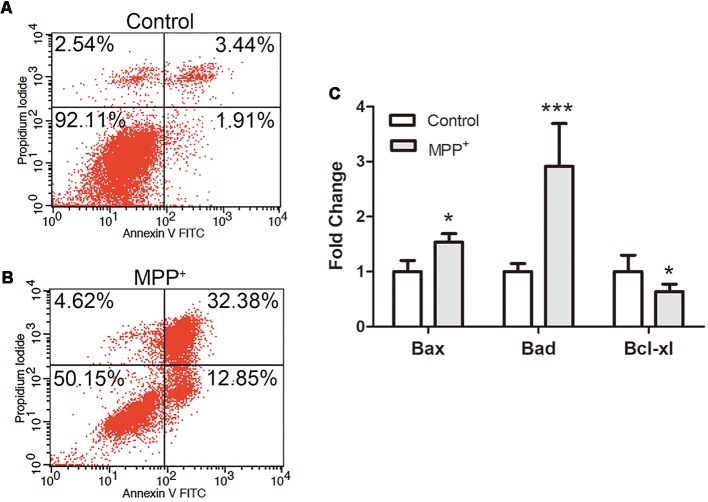
SH-SY5Y cell apoptosis and apoptosis relative genes expression analysis after treated with MPP^+^. **(A,B)** Cells were stained with FITC-labeled Annexin V and propidium iodide (PI) after treated without/with 1.73 mM MPP^+^ for 48 h, and analyzed by flow cytometry using FL1 (Annexin V) and FL2 (PI) channels. The values are shown in the lower left, lower right, upper right, and upper left quadrants of each panel represent the percentage of viable (Annexin V- PI-), early apoptosis (Annexin V^+^ PI^−^), late apoptosis (Annexin V^+^ PI^+^) and necrotic cells (Annexin V^−^ PI^+^), respectively. **(C)** The mRNA expression of *Bad*, *Bax*, and *Bcl-xl* was detected by qRT-PCR. Values were normalized to the values of control. Histograms are mean expression from three to five biological replicates. Error bars represent SD. *p < 0.05 and ***p < 0.001 compared with control.

In order to confirm the effect of inflammation, ROS and ΔΨm loss on cell apoptosis, SH-SY5Y cells were stained with AnnexinV-FITC and PI, and apoptosis was analyzed by flow cytometry. In control group, most of the cells remained alive (90% ± 3.1%), and the apoptotic cell population accounted for 5% ± 0.4% (Figure 4A). After treated with 1.73 mM MPP^+^ for 48 h, alive cells significantly decreased (51% ± 3.2%), while early and late apoptosis cells populations were both increased significantly (Figure 4B). The early and late apoptosis populations were 13% ± 0.41% and 31.89 ± 1.37%, respectively.

To further evaluate the apoptosis mechanism, the expression of apoptosis relative genes was analyzed by qRT-PCR. The results showed that after treated with MPP^+^, anti-apoptosis gene *Bcl-xl* was significantly down-regulated (0.73 ± 1.13), and pro-apoptosis genes *Bax* (1.34 ± 0.15) and *Bad* (2.92 ± 0.77) were significantly up-regulated (Figure 4C).

### MenSCs-CM Attenuates MPP^+^-Induced Cell Viability Reduction

**Figure 5:**
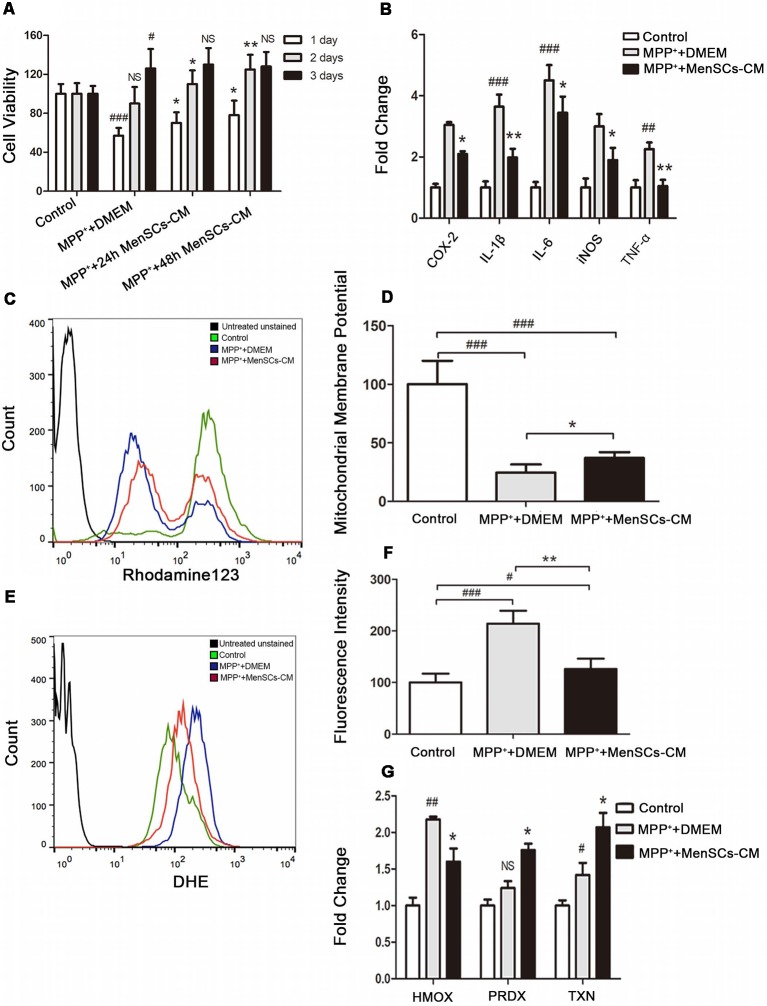
The effect of MenSCs-CM on cell viability, inflammation, mitochondrial membrane potential and ROS. **(A)** Cell viability was measured after culture with 24 h/48 h MenSCs-CM for different days. **(B)** The mRNA expression of *COX-2*, *IL-1β*, *IL-6*, *inducible nitric oxide synthase (iNOS)*, and *TNF-α* was detected by qRT-PCR. **(C)** Mitochondrial membrane potential was detected by Rhodamine123 staining using flow cytometry. **(D)** Statistical analysis of flow cytometry result in **(C)**. **(E)** ROS level was detected by DHE staining using flow cytometry. **(F)** Statistical analysis of flow cytometry result in **(E)**. **(G)** The mRNA expression of *HMOX-1, PRDX-1* and *TXN* was detected by qRT-PCR. All Histograms were mean expression from three to five biological replicates. Values were normalized to the values of control. Error bars represent SD. Comparisons among three groups were analyzed by one-way ANOVA and Bonferroni’s *post hoc*. ^#^*p* < 0.05, ^##^*p* < 0.01, and ^###^*p* < 0.001 compared with control group.NS, no significant difference compared with control group. *p < 0.05 and **p < 0.01 compared with MPP^+^+DMEM group.

MPP^+^-treated SH-SY5Y were cultured with 24 h and 48 h collected MenSCs for 1 day, 2 days, and 3 days, respectively. For control and MPP^+^+DMEM group, only DMEM basic medium was added. As shown in Figure 5A, after 1 day culture, although the cell viability of SH-SY5Y cells can restore from 50% to 57% in MPP^+^+DMEM group, 24 h and 48 h collected MenSCs-CM could help to increase cell viability to 70% and 78%, respectively, and there was a significant difference between MPP^+^+DMEM group and MPP^+^+24 h/48 h MenSCs-CM group (*p < 0.05). After 2 days culture, 24 h and 48 h MenSCs-CM could help to increase cell viability to 110% and 125%, respectively, and the cell viability was greatly significant difference between MPP^+^+48 h MenSCs-CM group and MPP^+^+DMEM group (**p < 0.01). After 3 days culture, the difference between MPP^+^+DMEM group and MPP^+^+24 h/48 h MenSCs-CM group became no significant (*p* > 0.05). So, the condition of 48 h-collected MenSCs-CM and cultivating with SH-SY5Y cells for 2 days was used for the following studies.

### MenSCs-CM Down-Regulates Pro-inflammation Cytokines Expression

Inflammation is an important inducer of cell death. To detect whether MenSCs-CM can decrease pro-inflammation factors expression, qRT-PCR was performed. We found that MenSCs-CM could decrease the gene expression of inflammation cytokines *COX-2*, *IL-1β*, *IL-6*, *iNOS*, and *TNF-α* (Figure 5B). So MenSCs-CM exists protective effect against MPP^+^ induced cytotoxicity *via* anti-inflammation.

### MenSCs-CM Promotes SH-SY5Y Survival *via* Restoring ΔΨm

The protective function of MenSCs-CM against MPP^+^-induced cytotoxicity was also effective in SH-SY5Y cells through restoring abnormal changes in mitochondrial membrane potential (Figures 5C,D). Figure 5C revealed that the ΔΨm of most SH-SY5Y cells significantly decreased in MPP^+^+DMEM group and only minority of cells had the same ΔΨm with cells in control group. While in MPP^+^ + MenSCs-CM group, there were more cells having similar ΔΨm with control group cells, and the number of cells lost ΔΨm decreased. There existed a significant difference between MPP^+^ + MenSCs-CM group and MPP^+^+DMEM group (*p < 0.05).

### MenSCs-CM Suppresses the Generation of ROS

Peak shifting was clearly observed in Figure 5E. After DHE staining, cells in control group had the lowest fluorescence intensity, which indicated lowest ROS level (Figures 5E,F). And ROS in MPP^+^+DMEM group was the highest. MenSCs-CM could significantly reduce ROS generation compared with MPP^+^+DMEM group (**p < 0.01), although still higher than control group (^#^*p* < 0.05). As shown in Figure 5G, after removing MPP^+^ drug and cultivation with DMEM medium for 2 days, the expression of *PRDX-1* and *TXN* increased to 1.24 ± 0.09 (^#^*p* > 0.05 vs. control) and 1.42 ± 0.16 (^#^*p* < 0.05 vs. control), respectively. If SH-SY5Y cells were cultured with MenSCs-CM after MPP^+^ drug treatment, mRNA expression level of *PRDX-1* and *TXN* increased to 1.76 ± 0.08 (*p < 0.05 vs. MPP^+^+DMEM group) and 2.07 ± 0.2 (*p < 0.05 vs. MPP^+^+DMEM group) respectively. The expression of anti-oxidant gene *HMOX-1* in MPP^+^+DMEM group was still greatly higher than control group (2.18 ± 0.036 vs. 1 ± 0.11, ^##^*p* < 0.01). And after cultured with MenSCs-CM, the expression of *HMOX-1* was decreased to 1.6 ± 0.18, which had a significant difference from MPP^+^+DMEM group (*p < 0.05).

### MenSCs-CM Promotes Neuronal Survival *via* Reducing Cell Apoptosis

**Figure 6:**
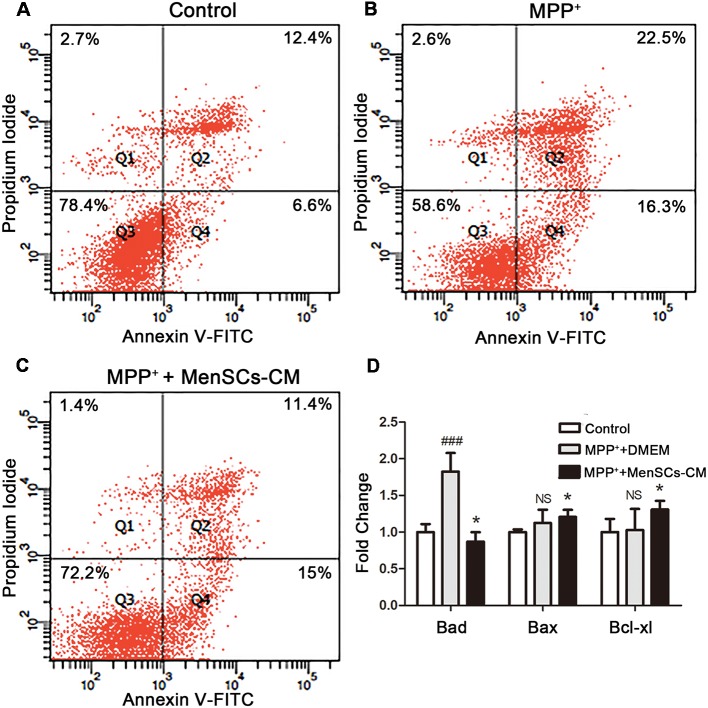
SH-SY5Y cells apoptosis and the expression profiles of apoptosis-related genes. **(A–C)** Apoptosis was detected by Annexin V/PI staining using flow cytometry. **(D)** The mRNA expression of *Bad*, *Bax*, and *Bcl-xl* was detected by qRT-PCR. Values were normalized to the values of control. Data were represented as mean ± SD from 3 to 5 biological replicates. Comparisons among 3 groups were analyzed by one-way ANOVA and Bonferroni’s *post hoc*. ^###^*p* < 0.001 compared with control group. *p < 0.05 compared with MPP^+^+DMEM group.

The potential protective effect of MenSCs-CM on MPP^+^-induced cell apoptosis was detected *in vitro*. For control group, around 78.4% cells remain viable (Figure 6A), which was lower than viable cell number (92.11%) shown in Figure 4A. The increasing of apoptosis is understandable because of lack nutrition.

For MPP^+^+DMEM group, after treated with MPP^+^ for 48 h, DMEM medium without FBS was added and cultured for another 2 days. Although the number of viable cells increased from about 50.15% to 58.6%, the expression level of pro-apoptosis genes *Bad* (1.12 ± 0.18) and *Bax* (1.89 ± 0.19) were still higher than control group (Figures 6B,D). Besides, the expression level of anti-apoptosis gene *Bcl-xl* increased to 1.03 ± 0.29 compared with control, but there was no significant difference (*p* > 0.05, Figure 6D).

For MPP^+^+MenSCs-CM group (Figure 6C), after cultured with MenSCs-CM, the number of viable cells was 72.2%, 13.6% more than MPP^+^+DMEM group. Besides, MenSCs-CM can significantly reduce the number of cells in late apoptosis stage compared with MPP^+^+DMEM group. What’s more, as shown in Figure 6D, the mRNA expression fold change of *Bad* and *Bax* were both decreased (*p < 0.05), and meanwhile the expression level of *Bcl-xl* was increased compared with MPP^+^+DMEM group (*p < 0.05).

The results indicated that MenSCs-CM could rescue the reduction of cell viability caused by MPP^+^ treatment through acting against cell apoptosis by up-regulating anti-apoptosis gene *Bcl-xl* and down-regulating the expression of pro-apoptosis genes *Bad* and *Bax*.

### Protein Assay of Neurotrophic Factors in MenSCs-CM

**Figure 7:**
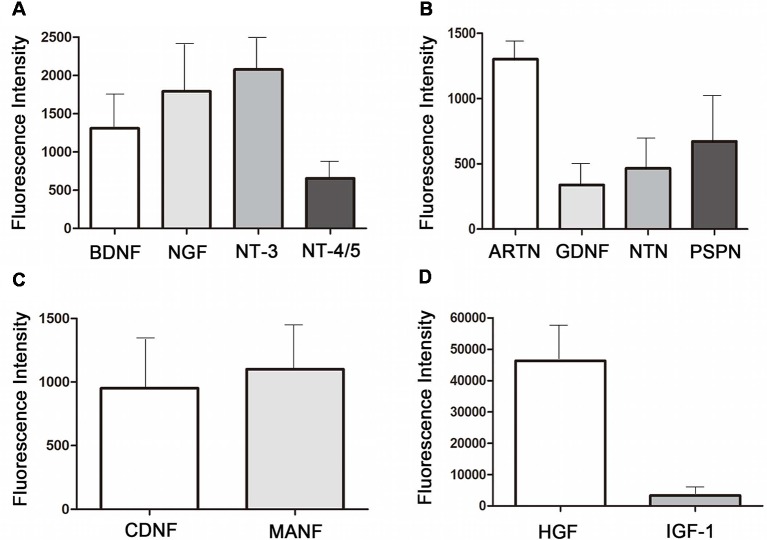
Neurotropic factors existing in the 48 h-collected MenSCs-CM.**(A)** The expression of neurotrophin family members in MenSCs-CM. **(B)** The expression of GFLs family members in MenSCs-CM. **(C)** The expression of novel dopaminergic neurotrophic factors in MenSCs-CM. **(D)** The expression of HGF and IGF-1 in MenSCs-CM. All histograms were mean expression from three biological replicates. Data were represented as mean ± SD.

To identify the neurotrophic factors released in the medium by MenSCs, protein array was performed. Twelve kinds of neurotrophic factors, including neurotrophin family, GDNF family of ligands (GFLs), novel dopaminergic neurotrophic factor family, HGF, and IGF-1 were detected. Figure 7A showed the expression of neurotrophin family members: BDNF, NGF, NT-3, and NT-4/5. Figure 7B showed the expression of GFLs members: ARTN, GDNF, NTN, and PSPN.

The expression of CDNF and MANF, which belonged to novel neurotrophic factors, was shown in Figure 7C. As shown in Figures 7A–D, among the 12 neurotrophic factors, HGF was the most highly expressed one, followed by IGF-1 and NT-3. The fluorescence intensity of GDNF was the lowest, followed by NTN and NT-4/5. Other factors were modest expressed in MenSCs-CM.

## Discussion

MenSCs failed to rescue damaged SH-SY5Y cell viability for 1–3 days in in-direct co-culture system (Supplementary Figure S1). No significant difference in cell viability was observed between MPP^+^+MenSCs group and MPP^+^+DMEM group (*p* > 0.05, Supplementary Figure S1). However, we found that MenSCs-CM significantly increased the cell viability of injured SH-SY5Y cells (Figure 2). MenSCs-CM may contain a higher concentration of MenSCs-secreted factors than that released by MenSCs during co-cultured with SH-SY5Y cells. This suggests that neurotropic factors secreted by MenSCs during co-culture were insufficient to exert therapeutic effect. Further studies need to perform to assess whether longer co-culture days or direct co-culture manner can contribute to detectable protection results.

In this article, we directly co-cultured MenSCs-CM with PD model for 1–3 days. There was a significant difference (**p < 0.01) between MPP^+^+48 h MenSCs-CM group and MPP^+^+DMEM group at day 2 (Figure 5A), but this effect was not observed on day 3 (*p* > 0.05). This is mainly due to insufficient nutrient to support cell growth for up to 3 days with only MenSCs-CM alone because MenSCs-CM contained only DMEM and components released by MenSCs, without FBS or other extra factors. Therefore, it is not suitable for culturing cells for more than 3 days.

MSCs not only secrete various soluble factors but also extracellular vesicles, which carry lipids, proteins, and nucleic acid (Vilaça-Faria et al., 2019). Among all the secreted vesicles, exosomes gained the most attention in recent years. In order to clarify whether exosomes alone could attenuate the cytotoxicity of SH-SY5Y cells induced by MPP^+^, different concentrations of MenSCs-CM derived exosomes (MenSCs-Exo) were prepared and cultured with injured SH-SY5Y cells for different days. Supplementary Figure S2 showed that both 100 μg/mL (10 μg) exosomes and 200 μg/mL (20 μg) exosomes significantly rescued MPP^+^-injured SH-SY5Y cells following treatment for 48 h or 72 h (*p < 0.05 or **p < 0.01 vs. MPP^+^+DMEM group). Besides, a higher concentration of exosomes at 200 μg/mL exerted better effect than 100 μg/mL exosomes. However, SH-SY5Y cell viability was reduced in group treated with 50 μg/mL exosomes for 48 h or 72 h (*p < 0.05 vs. MPP^+^+DMEM group). Furthermore, no significant difference in MPP^+^-injured SH-SY5Y cell viability after culturing with 5–200 μg/mL exosomes for 24 h or 5 μg/mL exosomes for 48 h/72 h (*p* > 0.05 vs. MPP^+^+DMEM group). Although 10 μg MenSCs-Exo increased MPP^+^-injured SH-SY5Y cell viability, it has to be isolated from at least 20 mL of MenSCs-CM, which in contrary, 100 μL of MenSCs-CM was enough to rescue the cell viability (Figure 5A), suggesting that MenSCs-CM is superior to MenSCs-Exo. This indicated that other components in MenSCs-CM may also play an important role in neuroprotection.

To highlight the function of soluble factors released by MenSCs, we prepared exosomes-deprived MenSCs-CM (EDM) and investigated the effect of EDM on SH-SY5Y cell viability. Supplementary Figure S3 showed that EDM significantly attenuates cell viability reduction of MPP^+^-injured SH-SY5Y after treated for 48 h or 72 h (*p < 0.05 vs. MPP^+^+DMEM group). There was no observable benefit on injured SH-SY5Y cell viability after treated with EDM for 24 h (*p > 0.05 vs. MPP^+^+DMEM group). Specifically, EDM increased SH-SY5Y cell viability from 58.29% ± 7.23 to 66.41% ± 8.29 at 24 h, 80.25% ± 8.09 to 105.14% ± 9.23 at 48 h, and 97.33% ± 6.37 to 119.47 ± 11.14 at 72 h. Compared with the results of MenSCs-CM treatment (Figure 5), the function of EDM is not as good as MenSCs-CM, suggesting that MenSCs-CM performed its neuroprotective function not only by secreted factors but also by exosomes.

Although the pathogenesis of PD is not completely revealed, mounting evidences prove that the dysregulation of immune system including innate and adaptive immunity plays an essential role in the pathogenesis of PD (Chen et al., 2018). The uncontrolled activated microglia releases many pro-inflammatory cytokines, including *COX-2*, *IL-1β*, *IL-6*, *TNF-α*, which provokes the onset of neuroinflammation and may trigger dopaminergic neurons death (Sun et al., 2013). Therefore, it is important to counteract pro-inflammatory cytokines for treating PD. Conditioned medium from bone marrow derived stem cells (BMSCs-CM) was reported to markedly reduce the expression of *IL-6*, *iNOS* and *TNF-α* in astrocytes cells derived from amyotrophic lateral sclerosis (ALS) transgenic mice (Sun et al., 2013). Fontanilla et al. (2015) reported that conditioned medium of human adipose derived stem cells (ADSCs-CM) increased ALS transgenic mouse lifespan by inhibiting microglia and astrocyte activation, reducing levels of phosphorylated p38, thus decreased inflammation and neuronal death in mice spinal cord. In this study, we mimicked the inflammatory process *in vitro* using neurotoxin MPP^+^. As expected, pro-inflammatory cytokines such as *COX-2, IL-1β*, *IL-6*, *iNOS*, *TNF-α* were markedly increased after exposure to MPP^+^. When MenSCs-CM was introduced in the culture system, there was a decrement pattern in all cytokines detected. Our study added value to the existing knowledge by showing that MenSCs-CM secreted anti-inflammation cytokines similar to that previously reported BMSCs-CM and ADSCs-CM in ALS disease. Further study on whether MenSCs-CM exerts an anti-inflammation effect on other neurodegenerative diseases apart from PD can be carried out.

The mechanisms by which MenSCs improved MPP^+^-induced cytotoxicity was carried out by performing protein assay on 12 different kinds of neurotrophic factors that played contribution to the protective function of MenSCs-CM. Neurotrophic factors referred here include neurotrophin family, GFLs, novel dopaminergic neurotrophic factor family, HGF, and IGF-1.

The neurotrophin family includes four types of factors: NGF, BDNF, NT-3, and NT-4/5, which promote dopaminergic neurons survival and nigrostriatal system regeneration (Rangasamy et al., 2010). NGF and BDNF are closely associated with the progression of PD, which were found to be decreased in PD patients (Lorigados Pedre et al., 2002; Rangasamy et al., 2010). NGF prevents the accumulation of ROS and inhibits cell apoptosis by Akt pathway and suppresses caspase-3 activation in dopaminergic PC12 cells (Shimoke and Chiba, 2001; Salinas et al., 2003). Likewise, BDNF could also decrease ROS in 6-OHDA-induced SH-SY5Y neuroblastoma cells by increasing glutathione reductase activity (Gu et al., 2009). Transplanting neural stem cells over expressing NT-3 showed better protective function than grafting neural stem cells alone (Rangasamy et al., 2010). NT-4/5 was reported to promote survival and morphological complexity of cultured dopaminergic neurons (Seiler et al., 2017). Altogether, the anti-oxidant and anti-apoptosis effect of MenSCs-CM on MPP^+^-injured SH-SY5Y cells may due to the presence of EGF, BDNF, NT-3, and NT-4.

GFLs family includes four structurally related trophic factors: GDNF, NTN, ARTN, and PSPN, which can restore and support the survival of several kinds of neurons including dopaminergic neurons (Sidorova and Saarma, 2016). Previous studies demonstrated that GDNF/NTN treatment can inhibit dopaminergic neurons loss and improve deficient behavior in PD model (Oiwa et al., 2002; Liu et al., 2007; Migliore et al., 2014; Tereshchenko et al., 2014). And stem cells overexpressing GDNF/NTN/PSPN could achieve better effect than cell graft alone (Ye et al., 2007; Deng et al., 2013; Yin et al., 2014; Hoban et al., 2015). Di Santo et al. showed combined used of GDNF and NT-4/5 synergistically protecting dopaminergic neurons (Di Santo et al., 2018). Administration of NTN/ARTN both significantly increased the survival and differentiation of ventral mesencephalon (Zihlmann et al., 2005). Despite reports on neuroprotective function of GFLs family members have been demonstrated, however, how they crosstalk in conditioned medium or which pathway they involve in to perform their function remain unclear.

Both CDNF and MANF belonged to the novel dopaminergic neurotrophic factor, which have the ability to protect dopamine neurons from death (Ren et al., 2013; Tereshchenko et al., 2014; Voutilainen et al., 2015; Garea-Rodríguez et al., 2016; Tang et al., 2017; Wang et al., 2017). For instance, after delivery of CDNF by adeno-associated virus type 2/8 into 6-OHDA treated rat striatum, it improved rat’s behavior deficit and restored TH positive dopaminergic neurons in substantial nigra and increased fiber density in striatum (Ren et al., 2013; Wang et al., 2017). In this study, we observed a larger amount of CDNF present in MenSCs-CM than GDNF. The protective role of CDNF in MenSCs-CM remains to be elucidated. Interestingly, in 6-OHDA induced PD model, CDNF treatment benefited rats motor function and increased TH^+^ fibers in striatum, but no effect on the number of TH^+^ cells in substantial nigra. Whereas, the function of MANF was totally opposite. The combined used of both factors exerted a synergistic effect on PD (Cordero-Llana et al., 2015).

HGF discovered in 1991, was demonstrated to play an important role in cell survival, tissue regeneration, and inflammation regulation in various injury and disease (Kitamura et al., 2011; Nakamura et al., 2011; Jeong et al., 2012; Ko et al., 2018; Li et al., 2018; Pang et al., 2018). It reduced the expression level of *IL-6*, *TNF-α*, and intercellular adhesion molecule-1 (*ICAM-1*) by regulating NF-kB signaling in pulmonary artery hypertension rat (Pang et al., 2018). Its application also promoted primate’s or rodent’s functional recovery in spinal cord injury by reducing the size of damaged parenchyma, decreasing glia scar formation, and increasing axon growth (Kitamura et al., 2011; Jeong et al., 2012). In our study, we showed that HGF was the most protein found in MenSCs-CM. However, it remained to be further explored whether it contributes to MenSCs-CM mediated neuroprotection in cell level PD model and what is the mechanism.

ADSCs-CM is well known for its therapeutic potential in neuron injury related diseases *in vivo* and *in vitro* (Wei et al., 2009; Zhao et al., 2009; Gu et al., 2013; Sun et al., 2013; Fontanilla et al., 2015). The neutralization of IGF-1 and BDNF in ADSCs-CM reduced the neuroprotective activity of ADSCs-CM in both *in vitro* and *in vivo* neuron injury models (Wei et al., 2009). Furthermore, ADSCs-CM was shown to protect glutamate-induced cerebellar granule neurons (CGN) death by down-regulation of activated caspase-3 and p38. Furthermore, inhibition of BDNF markedly weaken the anti-apoptosis function of ADSCs-CM *via* increasing caspase-3 activity but had no effect on phosphorylated p38. These suggested that ADSC-CM was better than BDNF treatment alone despite its neuroprotective ability. ADSC-CM may consist of other complementary activities that were responsible for blocking p38 activation (Zhao et al., 2009). Wei et al. compared the effect of ADSCs-CM, BDNF, and IGF-1 on hypoxia-ischemia-induced brain damage. Five gram BDNF and 50 g IGF were showed to promote neuron survival delivered by intracerebroventricular injection. And equal benefit could be achieved by administering ADSCs-CM, which have been found to contain only 1690pg IGF-1 and 33.5pg BDNF (Wei et al., 2009). With all of these evidences on neurotrophic factors found in MenSCs-CM that are beneficial to dopaminergic neurons, we concluded that all these neurotropic factors found in MenSCs-CM maybe function together as a neuroprotective mixture body. The neuroprotective effect of MenSCs-CM can be fully or partially abrogated through the neutralization of each factor.

In conclusion, we first demonstrated that MenSCs-CM can protect MPP^+^-induced cytotoxicity *via* reducing inflammation, oxidative stress, apoptosis and rescuing mitochondrial membrane potential. We showed that at least 12 different neurotrophic factors existing in the MenSCs-CM, which may contribute to the protective function of MenSCs-CM to treat PD cell model. However, it is still unclear how these 12 neurotrophic factors crosstalk with each other and which signal pathways they involved in to perform their protective function. Future studies will be focusing on the use of agonists of these 12 neurotrophic factors to neutralize the factors one by one to further confirm their roles in PD therapy. This research highlights the beneficial role of MenSCs-CM in treating PD and probably also for other neurodegenerative diseases.

## Author Contributions

All the authors provided important contributions and approved the final version of the manuscript. All authors were involved in conceiving and designing the experiments. HL performed the experiments and wrote the manuscript. BY, WN, NMY and JL contributed to technical guidance, troubleshooting, data analysis, and article revision.

## Conflict of Interest Statement

The authors declare that the research was conducted in the absence of any commercial or financial relationships that could be construed as a potential conflict of interest.
