# Supplementary material for: Conditioned Medium of Human Menstrual Blood-Derived Endometrial Stem Cells Protects Against MPP+-Induced Cytotoxicity in vitro
Source: Front Mol Neurosci. 2019 Apr 5;12:80. doi: 10.3389/fnmol.2019.00080 (PMC6460823; doi:10.3389/fnmol.2019.00080)
Supplement: Supplementary file 1 [file Data_Sheet_1.docx]

Supplementary Material

1. Supplementary table

**Supplementary table 1.** List of quantitative RT-PCR Primer Sequences

| **Gene name** | **Forward primer sequence (5' → 3')** | **Reverse primer sequence (5' → 3')** |
| --- | --- | --- |
| GAPDH | GTCTCCTCTGACTTCAACAGCG | ACCACCCTGTTGCTGTAGCCAA |
| IL-1β | ATGATGGCTTATTACAGTGGCAA | GTCGGAGATTCGTAGCTGGA |
| IL-6 | ACTCACCTCTTCAGAACGAATTG | CCATCTTTGGAAGGTTCAGGTTG |
| iNOS | TTCAGTATCACAACCTCAGCAAG | TGGACCTGCAAGTTAAAATCCC |
| TNF-α | CCTCTCTCTAATCAGCCCTCTG | GAGGACCTGGGAGTAGATGAG |
| COX-2 | CTGGCGCTCAGCCATACAG | CGCACTTATACTGGTCAAATCCC |
| HMOX-1 | AAGACTGCGTTCCTGCTCAAC | AAAGCCCTACAGCAACTGTCG |
| PRDX-1 | CCACGGAGATCATTGCTTTCA | AGGTGTATTGACCCATGCTAGAT |
| TXN | GTGAAGCAGATCGAGAGCAAG | CGTGGCTGAGAAGTCAACTACTA |
| Bad | CCCAGAGTTTGAGCCGAGTG | CCCATCCCTTCGTCGTCCT |
| Bax | CGAGAGGTCTTTTTCCGAGTG | GTGGGCGTCCCAAAGTAGG |
| Bcl-xl | CGGTACCGGCGGGCATTCAG | CGGCTCTCGGCTGCTGCATT |

1. Supplementary figures





**Supplementary Figure 1.** The effect of MenSCs on SH-SY5Y cell viability. MenSCs/DMEM was indirectly co-cultured with MPP^+^-injured SH-SY5Y cells for 24h, 48h, and 72h, respectively. Then cell viability was detected by Prestoblue and data was normalized by control group. Data was presented as mean ± SD. Comparisons between 2 groups were analyzed by Student-t test. NS: no significant difference.





**Supplementary Figure 2.** The effect of MenSCs-derived exosomes on SH-SY5Y cell viability. Different concentrations of MenSCs-Exo were added into MPP^+^-injured SH-SY5Y cells and cultured for 24h, 48h, and 72h, respectively. Then cell viability was detected by Prestoblue and data was normalized by control group. Data was presented as mean ± SD. Comparisons between 2 groups were analyzed by Student-t test. *p<0.05 and **p<0.01 compared with MPP^+^ + DMEM group; NS: no significant difference.





**Supplementary Figure 3.** The effect of exosomes deprived MenSCs-CM on SH-SY5Y cell viability. EDM was added into MPP^+^-injured SH-SY5Y cells and cultured for 24h, 48h, and 72h, respectively. Then cell viability was detected by Prestoblue and data was normalized by control group. Data was presented as mean ± SD. Comparisons between 2 groups were analyzed by Student-t test. *p<0.05 compared with MPP^+^ + DMEM group; NS: no significant difference.
